# HiCHap: a package to correct and analyze the diploid Hi-C data

**DOI:** 10.1186/s12864-020-07165-x

**Published:** 2020-10-27

**Authors:** Han Luo, Xinxin Li, Haitao Fu, Cheng Peng

**Affiliations:** 1 Hubei Key Laboratory of Agricultural Bioinformatics, College of Informatics, Huazhong Agricultural University, Wuhan, 430070 China; 2 Center for Life Sciences, School of Life Sciences, Yunnan University, Kunming, 650500 China

**Keywords:** Systematic bias, Hi-C, Diploid cell, HiCHap, 3D genome

## Abstract

**Background:**

In diploid cells, it is important to construct maternal and paternal Hi-C contact maps respectively since the two homologous chromosomes can differ in chromatin three-dimensional (3D) organization. Though previous softwares could construct diploid (maternal and paternal) Hi-C contact maps by using phased genetic variants, they all neglected the systematic biases in diploid Hi-C contact maps caused by variable genetic variant density in the genome. In addition, few of softwares provided quantitative analyses on allele-specific chromatin 3D organization, including compartment, topological domain and chromatin loop.

**Results:**

In this work, we revealed the feature of allele-assignment bias caused by the variable genetic variant density, and then proposed a novel strategy to correct the systematic biases in diploid Hi-C contact maps. Based on the bias correction, we developed an integrated tool, called HiCHap, to perform read mapping, contact map construction, whole-genome identification of compartments, topological domains and chromatin loops, and allele-specific testing for diploid Hi-C data. Our results show that the correction on allele-assignment bias in HiCHap does significantly improve the quality of diploid Hi-C contact maps, which subsequently facilitates the whole-genome identification of diploid chromatin 3D organization, including compartments, topological domains and chromatin loops. Finally, HiCHap also supports the data analysis for haploid Hi-C maps without distinguishing two homologous chromosomes.

**Conclusions:**

We provided an integrated package HiCHap to perform the data processing, bias correction and structural analysis for diploid Hi-C data. The source code and tutorial of software HiCHap are freely available at https://pypi.org/project/HiCHap/.

**Supplementary Information:**

The online version contains supplementary material available at 10.1186/s12864-020-07165-x.

## Background

The rapid development of Hi-C [1] and its derivatives [2–4] has revealed the hierarchical principle of chromatin three-dimensional (3D) organization. The original Hi-C work revealed that the chromatin was separated into A and B (active and inactive) compartments [1]. Several works further revealed the existence of topological domains [5–8], and the subsequent works found that the topological domains were also hierarchically organized by smaller domains [9–11]. The in situ Hi-C revealed that chromatin loop was another important layer in chromatin 3D organization [2]. The transcription factors, CTCF [12], Cohesin [13, 14] and ZNF143 [15], play important roles in mediating or stabilizing chromatin loops.

The 3D organization of homologous chromosomes can differ in some chromatin regions or at specific development stages. The chromatin loops in the H19 imprinted control region show significant differences between maternal and paternal chromosomes in human cell line GM12878 [2]. During X-chromosome inactivation, the active one and inactive one exhibit organizational differences [16–18]. The maternal and paternal chromosomes are also different in 3D organization at the early mouse embryogenesis, and these organizational differences gradually disappear at the later development stage [19, 20].

A few softwares, such as HiC-Pro [21] and Juicer [22], contained the module to construct diploid Hi-C contact maps, i.e., constructing contact matrices for two homologous chromosomes respectively, by using phased genetic variants. Recently, Tan et al. developed a pipeline to construct and analyze diploid Hi-C contact maps at the single cell level [23]. However, these softwares neglected the systematic biases in diploid Hi-C contact maps, especially the biased number of allele-assigned contacts caused by variable genetic variant density in the genome. In addition, few of the softwares could quantitatively perform the allele-specific analyses on different layers of chromatin 3D organization, including compartments, topological domains and chromatin loops. In this work, we proposed a novel strategy to correct the systematic biases in diploid Hi-C contact maps, and applied it to developing a software, called HiCHap, to process diploid Hi-C data for phased haplotypes. Our results show that the proposed correction strategy in HiCHap significantly improves the quality of constructed diploid Hi-C contact maps, which facilitates the diploid identification of compartments, topological domains and chromatin loops at various resolutions. HiCHap also supports data analysis on haploid Hi-C maps, i.e., analyzing Hi-C data without distinguishing homologous chromosomes.

## Implementation

### Overview

**Fig. 1:**
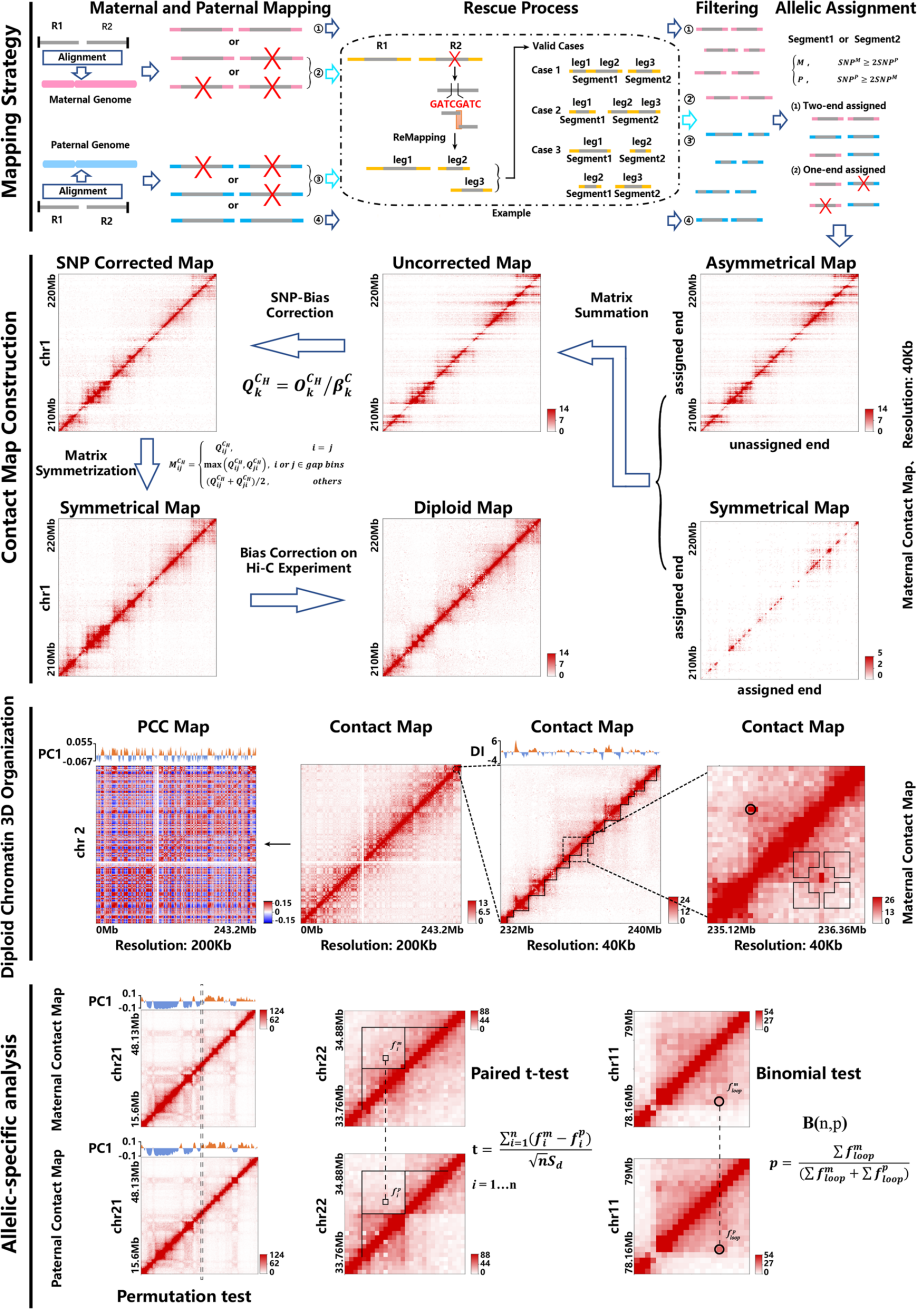
The workflow of HiCHap. Four modules are contained in HiCHap, including read mapping, contact map construction, identification of diploid chromatin 3D organization (compartment, topological domain and chromatin loop) and allele-specific testing on chromatin 3D organization

HiCHap is consisted of four modules: read mapping, contact maps construction, identification of diploid chromatin 3D organization (compartment, topological domain and chromatin loop) and allele-specific analysis (Fig. 1). Specifically, the paired-end reads are first mapped to maternal and paternal genomes which are built by using phased single nucleotide polymorphisms (SNPs). The unmapped end of a read is split into several parts according to the scanned ligation junction sites, and then all split parts are re-mapped to keep all available SNP information in the reads. The noisy reads are filtered and the kept ones are assigned to maternal or paternal reads according to their SNP information. Then the maternal and paternal contact matrices are constructed by using our proposed strategy of bias correction. The compartments, topological domains and chromatin loops are identified by using principal component analysis [1], directionality index based hidden markov model [5, 10] and HiCCUPS [2] with some modifications for diploid Hi-C contact matrices. Then the allele-specific compartments, topological boundaries and chromatin loops are tested. In following statement, haploid and diploid data processing refer to the processing procedure without and with distinguishing two homologous chromosomes in diploid cells.

### Hi-C read mapping

For diploid read mapping, all paired-end Hi-C reads were aligned to maternal and paternal genomes respectively by using Bowtie2 [24]. If one end of a Hi-C read was not mapped to maternal and paternal genomes, this end was scanned by the ligation junction sites. If no junction site existed in this unmapped end, the corresponding Hi-C read was discarded. Otherwise, this end was split in the junction sites and each part was mapped to maternal and paternal genomes again. The restriction-enzyme sites were used to determine the contact relationship among these split parts. Specifically, if two parts were in the same restriction fragment, they were considered to be in the same contact anchor (case 1 and case 2 in mapping strategy, Fig. 1). Occasionally, the split parts did not localize in the same restriction fragment due to complicated ligation procedure, and then two contacts were generated from one paired-end Hi-C read (case 3 in mapping strategy, Fig. 1). The mapped reads next underwent filtering by using the procedure in hiclib [25]. Finally, the obtained end was assigned to be maternal one if the number of maternally matched SNPs was larger or equal to the two times of the number of paternally matched SNPs in this end, and vice versa. If only one matched SNP existed in the end, this end was assigned to its matched parental genome. If no SNP was matched in this end, this end was considered to be unassigned. The haploid Hi-C read mapping was same as the diploid Hi-C read mapping except for two steps. First, the two ends of a Hi-C read were mapped to reference genome instead of maternal and paternal genomes. Second, no allele assignments were performed in haploid Hi-C read mapping.

### Matrix construction and bias correction

The haploid contact matrices were constructed by following previous pipeline [25]. The vanilla coverage (VC) normalization [1, 2] was used to correct biases to keep consistent with diploid contact matrix normalization in this work. However, the iterative correction strategy [25] was also provided in HiCHap.

Two types of diploid contact matrices were constructed. The first type of diploid contact matrices was constructed by using the pipeline of haploid matrix construction, except that only the two-end assigned contacts were used to construct maternal and paternal contact matrices. Since only a small proportion of contacts could be simultaneously assigned at two ends, these two-end-assigned contact matrices were very sparse (Supplementary figure 1A). The second type of diploid contact matrices was constructed by imputing the one-end-assigned contacts to improve data utilization. If two ends of a contact were in the same chromosome number, this contact was directly considered to be intra-haplotype contact due to the high proportion of intra-haplotype contacts calculated from two-end-assigned contact matrices (Supplementary figure 1B). If the two ends of a contact were in different chromosome numbers, this contact was imputed by following a previously proposed procedure [23].

To correct the allele-assignment bias caused by variable SNP density, the one-end-assigned contacts were placed in an asymmetrical way, in which the assigned end and unassigned end were placed in the row and column respectively. Then the symmetrical two-end-assigned contact matrices and the asymmetrical one-end-assigned contact matrices were summed (contact map construction, Fig. 1) to make use of all available data. Intuitively, some chromosomal bins were poor in the mapped contacts due to limited SNPs, and they were called gap bins in this work (Supplementary figure 2A). Let ${\left({O}_{ij}^{C_H}\right)}_{N_c\times {N}_c}$ denote the summed contact matrix for chromosome *C*, where the symbol *C*_*H*_ represents the maternal (*C*_*M*_) or paternal (*C*_*P*_) contact matrix and *N*_*c*_ denotes the number of chromosomal bins. The non-zero contact ratio for chromosomal bin *k* was defined as *r*_*k*_ = *K*/*N*_*C*_, where *K* is the number of non-zero contacts in the row vector ${O}_k^{C_H}=\left({O}_{k1}^{C_H},{O}_{k2}^{C_H},\cdots, {O}_{k{N}_C}^{C_H}\right)$. Let *r*^*t*^ denote the lower 25 percentile (Supplementary figure 3A) of all non-zero contact ratios $\left\{{r}_1,{r}_2,\cdots, {r}_{N_C}\right\}$, and the threshold of *r*^*t*^ was defined as $$ \mathrm{t}=\left\{\begin{array}{c}{r}^t,{r}^t\le 0.2\\ {}0.2,{r}^t>0.2\end{array}\right. $$ (Supplementary figure 3B). Then the bin *k* was defined as gap bin if *r*_*k*_ ≤ *t* in either maternal or paternal summed contact matrix. If chromosomal bin *k* was not gap bin, let ${f}_k^{C_M}={\sum}_{i=1}^{N_C}{O}_{ki}^{C_M}$ and ${f}_k^{C_P}={\sum}_{i=1}^{N_C}{O}_{ki}^{C_P}$ denote the summations of maternal and paternal contact frequencies respectively, and ${f}_k^C={\sum}_{i=0}^{N_C}{O}_{ki}^C$ denote the corresponding summation from haploid contact matrix ${\left({O}_{ij}^C\right)}_{N_c\times {N}_c}$. The allele-assignment ratio led by SNP density was defined as ${\alpha}_k^C=\left({f}_k^{C_M}+{f}_k^{C_P}\right)/{f}_k^C$. Then the SNP-bias correction factor for bin *k* was defined as $$ {\beta}_k^C=\left\{\begin{array}{cc}1,&\ if\ {\alpha}_k^c\ge {\alpha}_{max}^c\\ {}{\alpha}_k^c/{\alpha}_{max}^c,& if\ {\alpha}_k^c<{\alpha}_{max}^c\end{array}\right. $$, where ${\alpha}_{max}^C$ denotes the upper 80 percentile (Supplementary figure 3C) of all available allele-assignment ratios in chromosome *C* by excluding gap bins. The row vector *k* in the summed contact matrix ${\left({O}_{ij}^{C_H}\right)}_{N_c\times {N}_c}$ was corrected by ${Q}_k^{C_H}={O}_k^{C_H}/{\beta}_k^C$. The inter-haplotype contact frequencies for chromosomal bin *k* were corrected in the same way by using the SNP-bias correction factor ${\beta}_k^C$.

Next the corrected matrix was symmetrized by: $$ {M}_{ij}^{C_H}=\left\{\begin{array}{cc}{Q}_{ij}^{C_H},& i=j\\ {}\max \left({Q}_{ij}^{C_H},{Q}_{ji}^{C_H}\right),& i\  or\ j\in gap\  bins\\ {}\left({Q}_{ij}^{C_H}+{Q}_{ji}^{C_H}\right)/2,& others\end{array}\right. $$. Since many entries in this symmetric matrix exhibited zero or nearly zero values, we primarily adopted VC normalization [1, 2] to robustly reduce the biases caused by other sources: ${M}_{ij}^{\prime }={M}_{ij}^{C_H}/\left(\sqrt[3]{W_i^2}\times \sqrt[3]{L_j^2}\right)$, where *W*_*i*_ and *L*_*j*_ are the summations of *i*^*th*^ row and *j*^*th*^ column in the symmetric matrix $\left({M}_{ij}^{C_H}\right)$. However, the iterative correction [25] was also provided for diploid contact matrices in HiCHap. Finally, the average frequency of bias-corrected contact matrix was recalibrated to the average frequency of original contact matrix by: ${f}_{ij}=\left( ave\left({O}_{ij}^{C_H}\right)/ ave\left({M}_{ij}^{\prime}\right)\right)\times {M}_{ij}^{\prime }$, where *ave*() denotes the average value of all contact frequencies in the matrix. The inter-haplotype contact matrices were symmetrized, normalized and recalibrated in the similar way by using inter-haplotype contact frequencies instead of intra-haplotype contact frequencies.

### Identification of chromatin 3D organization

For the corrected haploid contact matrices, compartments were identified by using the principal component analysis [1]. The first, second and third principal components were selected as candidates to determine compartment A and B. For principal component *i*, let ${\mu}_A^i$ and ${\mu}_B^i$ denote the average strengths among the same kind of compartmental bins (A and B) respectively, in which the correlation matrices were used for strength calculations. Similarly, let ${\mu}_{AB}^i$ denote the average strength among compartment A and compartment B. Then the principal component with the maximum value of $\left({\mu}_A^i+{\mu}_B^i\right)/2-{\mu}_{AB}^i$ was selected as the final one for compartment identification. The topological domains were called by using the hidden markov model with modified directionality index (DI) [9, 10], in which three Gaussian mixed distributions were used in this work. The chromatin loops were called by using the modified HiCCUPS algorithm proposed previously [15]. The minor modification in this work was that the called significant contacts were removed from loop calling if their contact frequencies were below the corresponding median frequencies calculated from all contacts with the same genomic distance.

As for the corrected diploid contact matrices, the maternal and paternal compartments, topological domains and chromatin loops were identified in similar procedures to the haploid ones. However, different kinds of gaps in these sparse contact matrices should be taken into consideration to reduce the false-positive results. For a given chromosomal bin, if less than 5% intra-haplotype contacts showed non-zero contact frequencies in either maternal or paternal contact matrices, the row and column of this bin, called compartment gap in this work, were removed in compartment calculation (Supplementary figure 2B). For a given maternal (and paternal) contact matrix, the first, second and third principal components were used to calculate the Pearson Correlation Coefficients (PCCs) to the PC1 derived from haploid contact matrix respectively. The principal component with largest absolute PCC was selected as final one, and it was multiplied by the corresponding PCC sign to keep the identified compartment consistent. For a selected chromosome, if all PCCs were smaller than given threshold (0.7 in this work), the exception was reported. In boundary calling, if less than 80% local intra-haplotype contacts were non-zero contacts in the given window size, this chromosomal bin, called boundary gap in this work, was removed in calculation (Supplementary figure 2C). For an initially called boundary, if there existed 3 or more boundary gaps in upstream 7 bins, this boundary was not considered to be the end of topological domain. If there existed 3 or more boundary gaps in downstream 7 bins, this boundary was not considered to be the start of topological domain. If more than one third bins between two neighbor boundaries were boundary gaps, the chromatin region between these two boundaries was not considered to be a domain due to the potential existence of another boundary in these gaps. For a given contact, if both anchors were localized in the gap bins, this contact was removed in loop calling. If any one of its four neighbor contacts showed zero value (called loop gap in this work), this contact was also removed in loop calling (Supplementary figure 2D). To further remove the incredible loops, the initially called loops were weighted by ${S}_l={f}_{ij}^l\times \left(-{\log}_{10}q\right)$, where ${f}_{ij}^l$ denotes the contact frequencies of the initially called loops and *q* denotes the weighted q-values generated in loop calling. The loops with 15% lowest scores were further removed. Altogether, these empirical parameters in removing different types of gaps can be optionally set in HiCHap.

### Testing on allele-specific chromatin 3D organization

For maternal and paternal compartments, the chromosomal bins with changed compartmental signs were selected as initial set. Since these compartmental transitions could be generated from biological variations (Supplementary figure 4), the permutation test was used to select reliable compartmental transitions. Specifically, for the transitioned bins, the entry values in both maternal and paternal eigenvectors were selected to build the maternal and paternal entry sets respectively. Randomly selected one value from maternal entry set and one value from paternal entry set, and calculated the value difference by maternal entry value minus paternal entry value. Repeated this calculation to obtain enough values to draw the empirical distribution. For each transitioned bin in initial set, the matched entry-value difference between maternal and paternal eigenvectors was used to calculate the *p*-value by using the empirical distribution.

The topological boundaries from haploid, maternal and paternal contact matrices were merged in testing allele-specific boundaries. The boundaries from three kinds of contact matrices were aligned by using the threshold of 3 bins. For the exactly aligned boundary, the insulation score was calculated for each contact in the lower triangle of a 10 × 10 square window (Allele-specific analysis in Fig. 1). If more than 70% contacts exhibited zero values in either maternal or paternal window, the boundary was removed from calculation. Then the contact insulation scores between maternal and paternal windows were paired to calculate the *p*-value by using the paired t-test. If maternal and paternal boundaries were aligned but without exactly same position, the genomic positions of maternal and paternal boundaries were used for p-value calculations respectively. The lower p-value in the two calculations was used for the aligned boundary. If only maternal or paternal boundary was called, the genomic position of the called boundary was used in the calculation. If both maternal and paternal boundaries were not called due to algorithmic sensitivity, the position of haploid boundaries was used for calculation.

The haploid chromatin loops anchored in the aforementioned gap bins were removed in allele-specific testing. The haploid, maternal and paternal chromatin loops were aligned by using previously proposed method [15]. The binomial distribution B(*n*, *p*) was used to calculate the *p*-value for each aligned chromatin loop, where $\mathrm{p}=\sum {f}_{loop}^m/\left(\sum {f}_{loop}^m+\sum {f}_{loop}^p\right)$, and ${f}_{loop}^m$ and ${f}_{loop}^p$ denote the contact frequencies of maternal and paternal chromatin loops respectively. If the maternal and paternal chromatin loops were aligned but not matched exactly in position, the contact frequencies in their own positions were used for calculation. If only maternal or paternal chromatin loop was called, the two contact frequencies in the position of called chromatin loop were used for calculation. If both maternal and paternal chromatin loops were not called due to algorithmic sensitivity, the two contact frequencies in the position of haploid chromatin loop were used for calculation.

### Other calculations

To evaluate the parameter robustness in SNP-bias correction in HiCHap (Supplementary figure 3), the matrix similarities among different parameter values were calculated by using the reproducibility analysis from HiCRep [26]. The relationship between allelic contact number and the SNP number was calculated by using kernel density estimation with Gaussian kernel function. The PCCs between maternal (or paternal) principal components and haploid principal components were calculated chromosome-by-chromosome, and the maternal and paternal PCCs were combined in presentation. The PCCs for DI were calculated and analyzed in the similar way. The boundary insulation scores were calculated by following a previous method [27]. The minor modification was that the zero-value contacts were excluded from calculations to reduce the negative impact of different kinds of gaps. The averaged contact maps of maternally biased and paternally biased boundaries were also calculated by excluding zero-value contacts. The loop strength was calculated by following the previous pipeline [15] modified from aggregate peak analysis [2]. The averaged contact maps of maternally biased and paternally biased chromatin loops were calculated by excluding loop gaps. Since the constructed diploid contact matrices were sparse at high resolutions, the maternal and paternal chromatin loops were mainly called and analyzed at the 40 kb resolution if there was no explicit statement. Occasionally, the 20 kb resolution was used for comparisons in this work. To obtain enough number of allele-specific chromatin 3D organizations for functional analyses, the allele-specific compartments with *p*-values smaller than 0.05 and the maternal/paternal PC entry ratios (or vice versa) larger than 1.5 were selected, and the allele-specific chromatin loops with p-values smaller than 0.05 and the maternal/paternal contact ratios (or vice versa) larger than 1.5 were selected. The allele-specific boundaries with adjusted p-values smaller than 0.01 were selected for statistics.

In the ChIP-Seq data processing, the reads were aligned to maternal and paternal genomes respectively. The obtained read was assigned to be maternal one if the maternally matched SNPs outnumber the paternally matched SNPs, and vice versa. In the enrichment analysis on the allele-specific compartments, the maternally and paternally mapped ChIP-Seq reads were processed by using deepTools with RPKM normalization under 1 kb bin size [28], and the averaged RPKM was subsequently calculated by excluding zeros in each given compartmental bin at the 200 kb resolution. Then the read difference between maternal and paternal bins were calculated by using Wilcoxon signed rank test. In the correlation analysis, the CTCF peak with largest allelic ratio in the given chromatin loop (40 kb resolution) was selected for the correlation calculation, and the same procedure was used in the Rad21 peak analysis.

## Results

### Variable SNP density leading to systematic biases in diploid Hi-C data

**Fig. 2:**
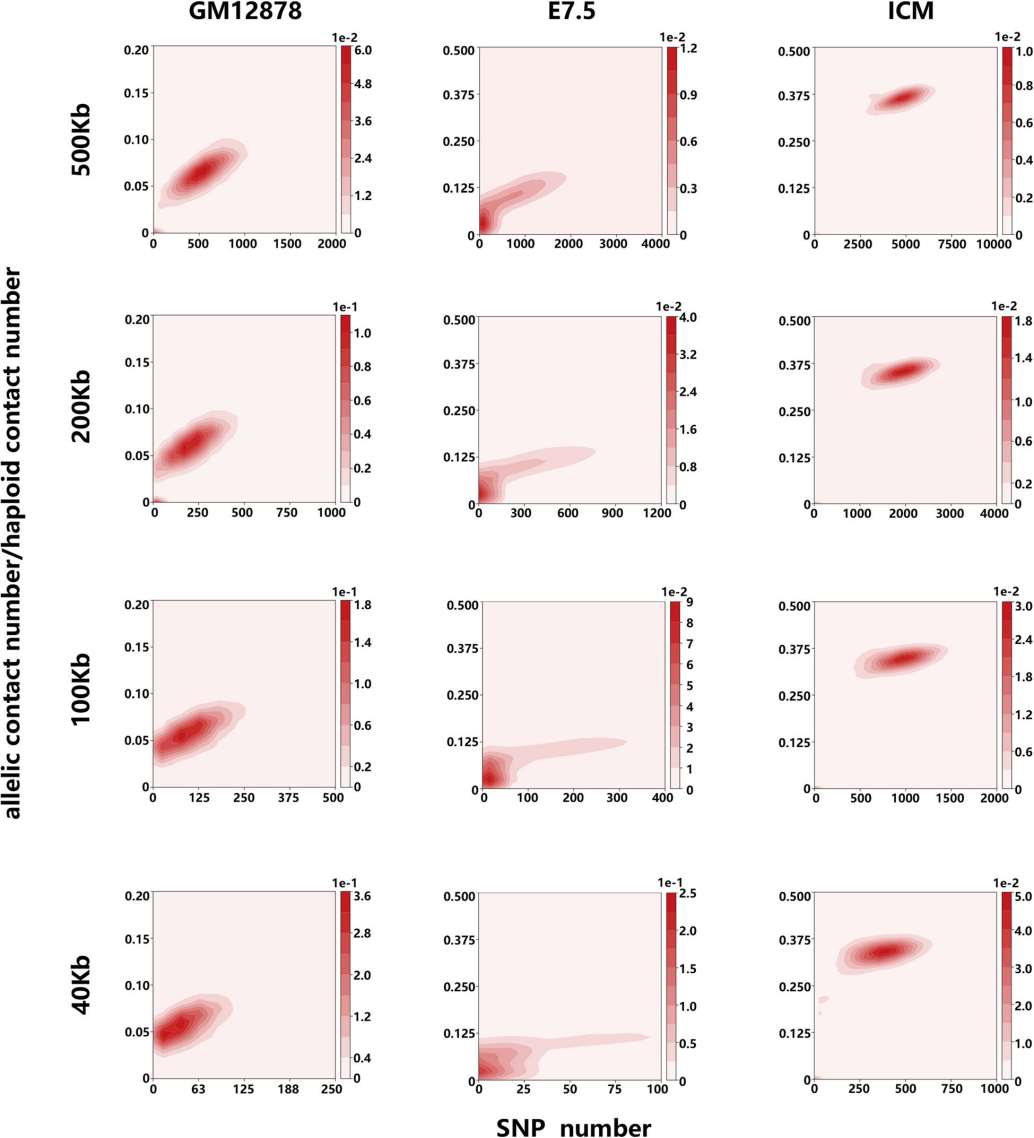
The relationship between the allelic contact number and the SNP number. The x-axis denotes the SNP number in all subfigures. The y-axis in all subfigures denotes the allele-assignment ratio by using the number of allele-assigned contacts dividing the number of haploid contacts

Previous study has shown that several steps of Hi-C experiment can lead to systematic biases in Hi-C data [29, 30]. Here we show an additional bias source in diploid Hi-C data caused by a specific step in data processing. Generally, the homologous Hi-C contacts are distinguished by using phased genetic variants, especially the single nucleotide polymorphisms (SNPs). However, the number of SNPs varies greatly among chromatin regions, implying that the chromatin regions with more phased SNPs can potentially be assigned more allelic contacts than those with less phased SNPs. To demonstrate the impact of SNP density on the number of assigned allelic contacts, we selected three Hi-C data sets to conduct the investigation, including the human cell line GM12878 [2], mouse embryos (day 7.5, denoted as E7.5) crossed by C57BL/6 and DBA/2 [20], and mouse inner cell masses from blastocyst (ICM) crossed by C57BL/6 and PWK/PhJ [19]. These three Hi-C data sets are available representatives with variable SNP density, among which the cell type ICM has the highest averaged SNP density and the cell type E7.5 exhibits highly variable SNP density in the genomes (Supplementary figure 5). We performed read mapping and allele assignment for these three Hi-C data sets by using phased SNPs, and constructed the asymmetrical matrices in which the rows and columns largely denoted the allele-assigned ends and allele-unassigned ends respectively for Hi-C contacts. We found that the allelic contact number showed complex nonlinear relationship to the SNP number, but the positive correlations could still be observed in most cases (Supplementary figure 6). Since the allelic contact number also depends on the available contacts in haploid contact maps, we used the allele-assignment ratio, the allelic contact number dividing the haploid contact number for a given chromatin bin, to perform the investigation to reduce the impact of other bias sources on analysis. Figure 2 illustrates that the cell line GM12878 and cell type ICM exhibit strong linear relationship between the allele-assignment ratio and the SNP number at various resolutions. The cell type E7.5 also exhibits a certain degree of linear relationship, but with much larger variations. These results suggest that the variable SNP density can lead to systematic biases in diploid Hi-C data.

### Bias correction in HiCHap

We developed a novel strategy to correct the systematics biases in diploid Hi-C data (contact map construction in Fig. 1), including the biases caused by variable SNP density and Hi-C experiment. To remove the allele-assignment bias caused by variable SNP density, the asymmetrical contact matrices were constructed by placing the allele-assigned ends in the rows and the allele-unassigned ends in the columns for those one-end assigned contacts. Though the number of two-end assigned contacts is much less than that of one-end assigned contacts, the symmetrical matrices constructed from the two-end assigned contacts were added to the corresponding asymmetrical contact matrices to make use of all available allele-assigned contacts. Since the ratio of allele-assigned contacts shows a certain degree of linear relationship to the SNP number (Fig. 2), each row of the obtained asymmetrical contact matrices was corrected by dividing a relative factor. Then the matrices were symmetrized by averaging the two entry values in corresponding rows. However, if one entry localized in the row with extremely low number of allele-assigned contacts, the other entry with higher contact frequency was used to construct symmetrical matrix to improve the data quality. After the first step of bias correction, the symmetrized contact matrices were further corrected by using the vanilla coverage (VC) normalization [1, 2] since this method could work robustly even in sparse diploid contact matrices. Other methods, such as iterative correction [25], could also be used in most cases, but the correction failure could happen occasionally in some sparse diploid contact matrices.

### Evaluation on the SNP-bias correction in HiCHap

Since previous softwares [21, 22] adopted different mapping strategies and did not provide bias correction for diploid Hi-C data, we used a pipeline, called SNP-biased method in this work, to fairly evaluate the effect of removing the allele-assignment bias caused by variable SNP density. The SNP-biased method adopted the same pipeline as HiCHap, except that the symmetrical matrices were directly constructed as previous softwares without directly removing allele-assignment bias. As for evaluation metrics, we did not directly measure the matrix similarities between diploid Hi-C contact maps and haploid Hi-C contact maps because the diploid Hi-C contact maps are quite sparse in many local regions at relatively high resolutions. We mainly used the principal components and DI derived from Hi-C contact maps to measure the organizational similarities among different kinds of contact maps since these metrics are popular methods to identify compartment and topological domain. We used the haploid Hi-C contact maps as controls in the evaluation since previous works have already shown that the low-resolution maternal and paternal Hi-C contact maps resemble the haploid Hi-C contact maps in overall [19, 20]. Our following calculations will further confirm the overall organizational similarities between diploid Hi-C contact maps and haploid Hi-C contact maps.

**Fig. 3:**
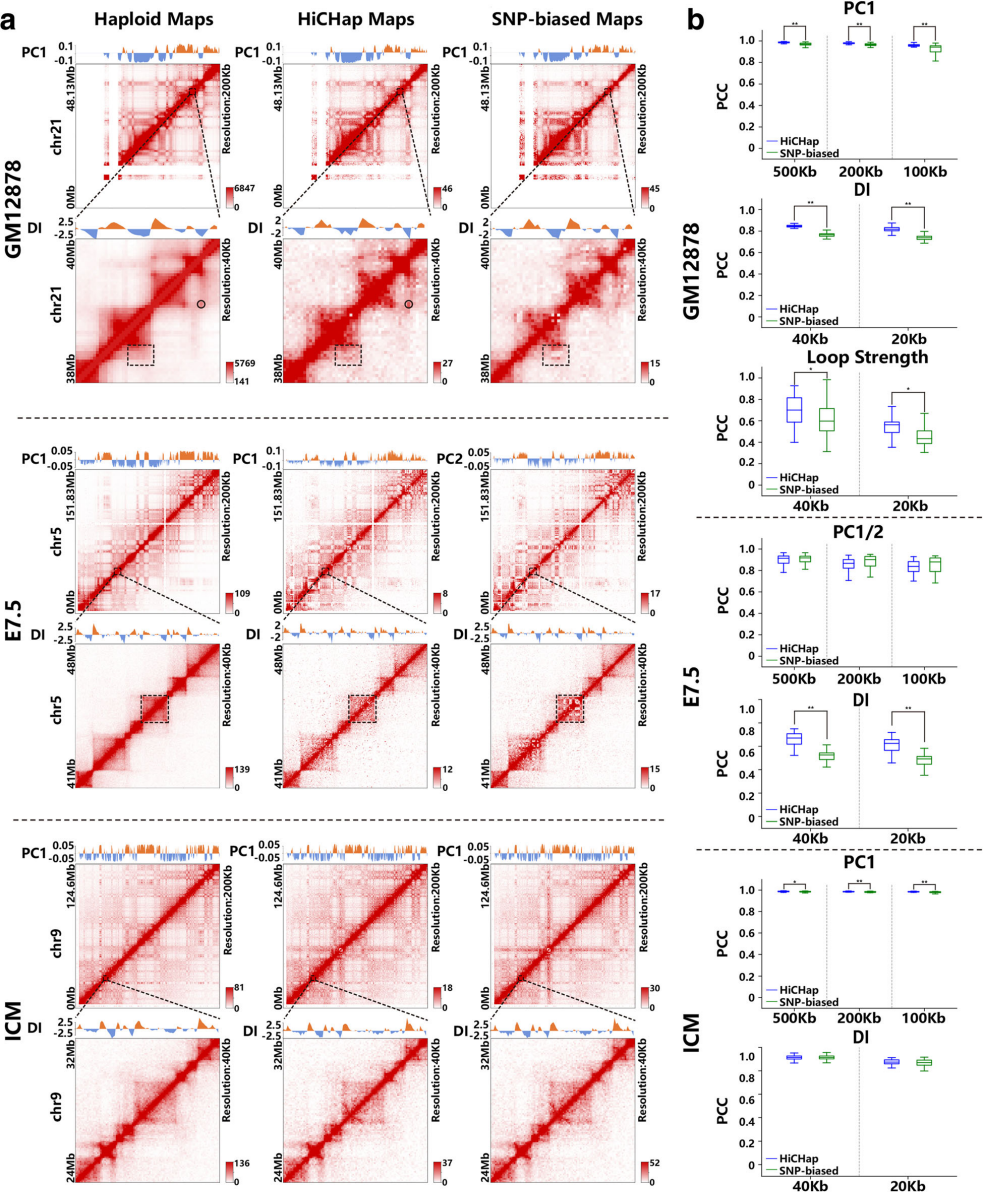
Evaluation of SNP-bias correction in HiCHap. (a) Examples showing the improved quality of HiCHap contact maps. Circles denote the called chromatin loop, and dashed rectangles show the map differences in local regions between HiCHap contact maps and SNP-biased contact maps. (b) Genome-wide evaluations (* 0.01 ≤ *p* < 0.05, ** *p* < 0.01). The boxplots are drawn by combining maternal and paternal PCCs. In cell type E7.5, the PC2 is selected for five chromosomes and the PC1 is selected for the rest chromosomes in HiCHap contact maps, whereas the PC2 is selected for all chromosomes in SNP-biased contact maps

Figure 3 illustrates that HiCHap contact maps outperform SNP-biased contact maps by using haploid contact maps as controls. Compared to the PC1 derived from SNP-biased contact map, the PC1 derived from HiCHap contact map shows higher similarity to that derived from haploid contact map in cell line GM12878, especially at the end part of chromosome (Fig. 3a). In cell type E7.5, it is also the PC1 derived from HiCHap contact map resembling that derived from haploid contact map, but it is the second principal component (PC2) derived from SNP-biased map resembling the PC1 derived from haploid contact map (Fig. 3a and Supplementary figure 7). The order change of principal components indicates that the highly variable SNP density in cell type E7.5 leads to substantial allele-assignment biases which cannot be corrected by VC normalization. In cell type ICM, the PC1 derived from both HiCHap contact map and SNP-biased contact map well resembles that derived from haploid contact map. As for DI, HiCHap contact maps outperform the SNP-biased contact maps in many local regions in cell line GM12878 and cell type E7.5. As shown in Fig. 3a, the HiCHap contact map exhibits stronger contact frequency than SNP-biased contact map in the given chromatin loop in cell line GM12878, resulting in the successful loop calling in HiCHap contact map but failed loop calling in SNP-biased contact map by using the same method. In addition, the HiCHap contact maps in cell line GM12878 and cell type E7.5 exhibit much smoother contact frequencies in many local regions, resembling the haploid contact maps better. However, the HiCHap contact map and SNP-biased contact map do not show obvious differences in the cell type ICM.

We also performed genome-wide evaluation on SNP-bias correction in HiCHap (Fig. 3b). Except the principal components and DIs, the chromatin loops in cell type GM12878 called at 5 kb resolution in the original in situ Hi-C paper [2] were also used for loop strength analysis. The cell types E7.5 and ICM were excluded from loop analysis since it is difficult to perform reliable loop calling in these two low-input Hi-C data sets. The chromosome X was also excluded from all kinds of statistics. As for principal component, the HiCHap contact maps outperform SNP-biased contact maps to some extent in cell line GM12878 and cell type ICM, though the PC1 derived from both types of contact maps shows quite high PCCs to the PC1 derived from haploid contact maps at different resolutions. In cell type E7.5, the PC2 but not the PC1 derived from SNP-biased contact maps shows highest PCCs to the PC1 derived from haploid contact maps in all chromosomes. At the 40 kb and 20 kb resolutions, the DIs derived from HiCHap contact maps show significantly higher PCCs than those derived from SNP-biased contact maps in cell line GM12878 and cell type E7.5. And no significant DI difference is observed in cell type ICM. As for chromatin loop, the same trend is observed in the cell line GM12878. We next identified compartments and topological boundaries in all three data sets and chromatin loops in cell line GM12878 from haploid contact maps, HiCHap contact maps and SNP-biased contact maps, and the results show the same trends as correlation analyses (Supplementary figure 8). Altogether, our results show that the SNP-bias correction in HiCHap can significantly improve the quality of reconstructed contact matrices at various resolutions in different cell types, which further facilitates the diploid identification of compartment, topological domain and chromatin loop.

### Allele-specific analyses on compartment, topological domain and chromatin loop

**Fig. 4:**
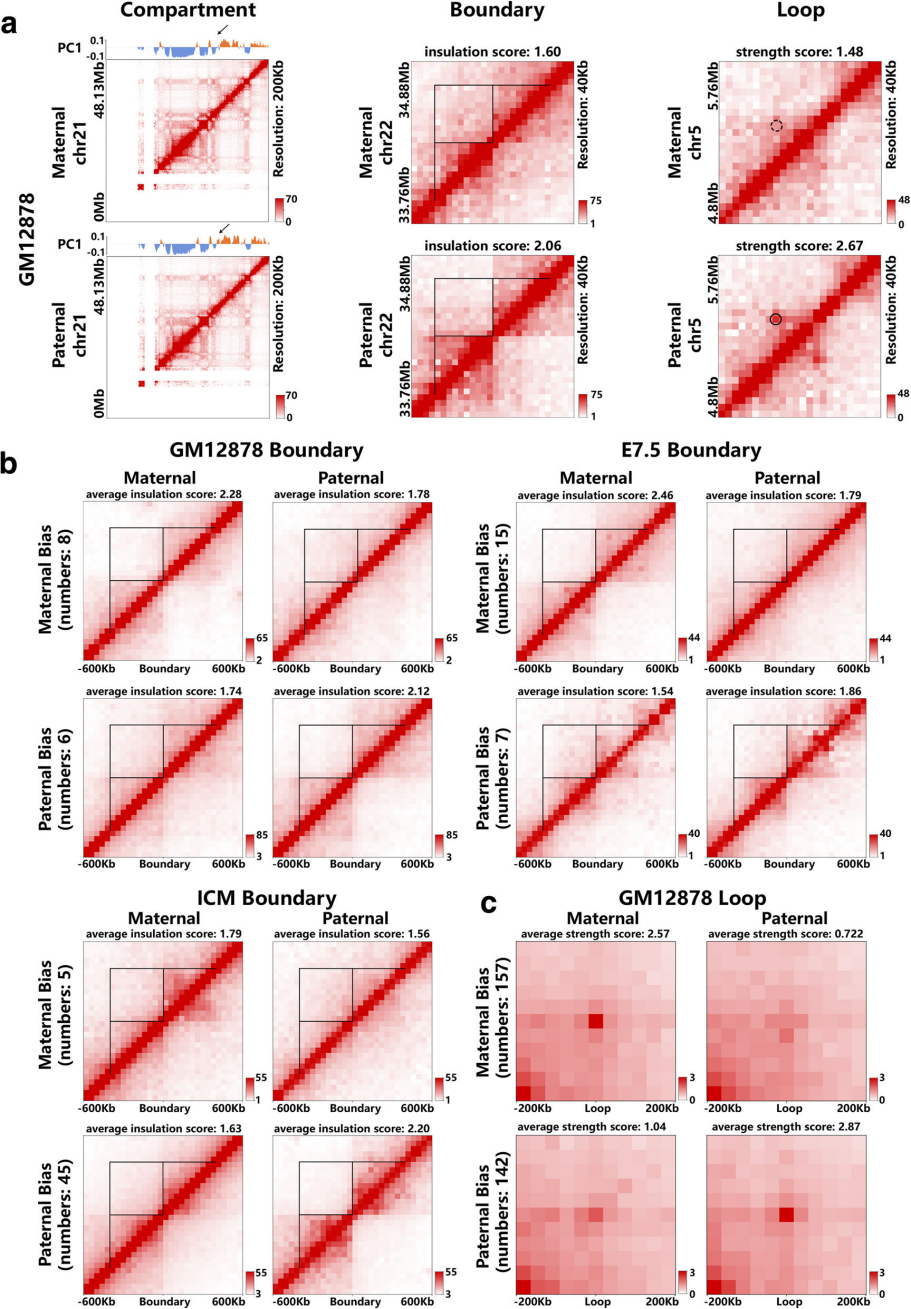
Allele-specific chromatin 3D organization. (a) Examples in cell line GM12878. Arrow points to the allele-specific compartment. Circle represents the success of loop calling in paternal contact map and dashed circle denotes the failure of loop calling in maternal contact map. (b) Averaged contact maps for maternally and paternally biased boundaries in cell line GM12878, cell type E7.5 and cell type ICM. (c) Averaged contact maps for maternally and paternally biased chromatin loops in cell line GM12878. The norm aggregate peak analysis was used for presentation

We next performed allele-specific analysis on compartment, topological boundary and chromatin loop by using HiCHap. As shown in Fig. 4a, the allele-specific chromatin 3D organization detected by HiCHap does exhibit contact differences between maternal and paternal contact maps in cell line GM12878. We then calculated the averaged contact matrices in the same allelic direction for each data set. In all three data sets, maternally and paternally biased boundaries exhibit higher insulation scores in the maternal and paternal contact maps respectively (Fig. 4b), consistent with the expected allelic directions. The same trends hold for maternally and paternally biased chromatin loops in cell line GM12878 (Fig. 4c). We further performed enrichment and correlation analyses on these allele-specific chromatin 3D organizations. The results show that the allele-specific compartments are associated with the allelic biases of epigenomic signals, such as active signal H3K4me1 (Supplementary Figure 9), and the allele-specific chromatin loops are positively correlated with the allelic biases of CTCF and Rad21 binding sites (Supplementary Figure 10). These results show that HiCHap can identify the significant differences between maternal and paternal chromatin 3D organizations. However, further works are needed in the future to strictly validate these allele-specific chromatin 3D organizations and explore their biological functions.

## Conclusions

Previous methods on diploid Hi-C data processing neglected the systematic biases in the constructed maternal and paternal contact matrices, especially the allele-assignment bias caused by the variable SNP density. In this work, we proposed a novel strategy to correct these biases and applied it to developing HiCHap software to analyze diploid Hi-C data for phased haplotypes. Our results show that the additional correction on the allele-assignment bias caused by variable SNP density can significantly improve the quality of constructed maternal and paternal contact matrices at various resolutions, which indicates that the previous correction methods in haploid contact maps cannot completely eliminate this specific type of bias in diploid Hi-C data. In addition, except the SNP-bias correction by using asymmetrical matrices, HiCHap constructs symmetric matrices by placing the relatively better one of the two contacts if they are around defined gaps. This matrix-construction strategy can further smooth maps and sharpen chromatin loops. However, it should be noted that the benefit of SNP-bias correction is also impacted by the number of phased SNPs. If the SNPs are highly dense for given resolutions, such as the presented cases in cell type ICM, the diploid Hi-C contact maps derived from VC normalization alone show relatively similar results to those derived from the allele-assignment bias correction plus VC normalization. If there are few and even no mapped reads in the given chromatin bins due to limited SNPs, it is difficult and even impossible to reliably correct the biases in these bins. With the rapid increase of phased haplotypes, HiCHap can play more important roles in exploring the diploid chromatin 3D organization in the near future.

## Availability and requirements

Project name: HiCHap.

Project home page: https://pypi.org/project/HiCHap/

Operating system(s): CentOS.

Programming language: Python.

Other requirements: bowtie2, samtools and cooler.

License: GPLv3.

Any restrictions to use by non-academics: None.

## Supplementary Information

**Additional file 1 : Supplementary figure 1**. Two-end assigned contact matrices. (A) Examples showing the sparseness of two-end assigned contact matrices. The contact matrices in human cell line GM12878 are sparser than those in the E7.5 and ICM due to its lower SNP density. E7.5 and ICM denote the mouse embryos (day 7.5) crossed by C57BL/6 and DBA/2 and mouse inner cell masses from blastocyst crossed by C57BL/6 and PWK/PhJ respectively. (B) The intra-haplotype contacts dominate the two-end assigned contacts in all three data sets. **Supplementary figure 2**. Examples of different kinds of gaps diploid Hi-C contact maps defined in HiCHap. The heatmaps are from the cell line GM12878. (A) Gap bins defined in asymmetrical matrix. (B) Compartment gaps defined in compartment identification. PC1 denotes the first principle component. (C) Boundary gaps defined in boundary calling. (D) Loop gap defined in loop calling. The loop gap here is the zero-value contact around given circle. **Supplementary figure 3**. Evaluation on the parameter robustness in the SNP-bias correction. (A) Matrix similarities among different percentile values used in the definition of gap bin. (B) Matrix similarities among different threshold values used in the definition of gap bin. (C) Matrix similarities among different percentile values used in the definition of SNP-bias correction factor. For each parameter at given resolution, the diploid contact matrices were generated by using five different values, in which the default values for the other two parameters were used. The similarities among the five maternal contact matrices were calculated chromosome-by-chromosome by using HiCRep (see Materials and methods), and the X chromosomes were excluded from the calculations. The average values were shown in the subfigures. **Supplementary figure 4**. Replicate reproducibility for the selected eigenvectors in principal component analysis. Haploid reproducibility was calculated by using the first eigenvectors derived from two replicated haploid contact maps. The maternal and paternal reproducibility was calculated in the same way. In the cell line GM12878 and cell type ICM, the first eigenvectors were selected for all chromosomes. In cell type E7.5, the second eigenvectors were selected for five chromosomes and the first eigenvectors were selected for the rest ones. The light green points indicate that the two eigenvector entries of the same chromatin bin can exhibit the signed change between two biological replicates, suggesting the necessity to account for the intrinsic variations when calculating allele-specific compartments (or compartmental transitions). Actually, the variations are higher in maternal and paternal contact maps than those in haploid contact maps. PCC denotes Pearson correlation coefficient. **Supplementary figure 5**. The SNP density distributions on chromosomes at the 200 kb resolution. (A) to (C) show the results in the cell line GM12878, cell type E7.5 and cell type ICM respectively. Compared to GM12878 and E7.5, the cell type ICM has the highest SNP density and relatively balanced SNP number in each chromosomal bin. By contrast, the cell type E7.5 exhibits the most variable SNP density in chromosomal bins. **Supplementary figure 6**. The relationship between allelic contact number and SNP number in cell line GM12878, cell type E7.5 and cell type ICM. The allelic contact denotes the contact which is assigned to be maternal or paternal one. In all subfigures, the x-axis and y-axis represent SNP number and allelic contact number respectively. **Supplementary figure 7**. The examples showing the differences in the used principle component number between HiCHap contact maps and SNP-biased contact maps in the cell type E7.5 at 200 kb resolution. PC1 and PC2 denote the first and second principle component respectively. **Supplementary figure 8**. The consistence of compartments, topological boundaries and chromatin loops among haploid contact maps, HiCHap contact maps and SNP-biased contact maps. (A) Compartment. Compared to SNP-biased contact maps, the A/B compartments derived from HiCHap contact maps show much better consistence with those derived from haploid contact maps in cell types GM12878 and ICM. In cell type E7.5, the consistence level is comparable between HiCHap contact maps and SNP-biased contact maps. (B) Boundary. The boundaries derived from HiCHap cotant maps show much better consistence with those derived from haploid contact maps in cell types GM12878 and E7.5, and comparable consistence in cell type ICM. (C) Chromatin loop. The trend in chromatin loop is similar to those in compartment and boundary in cell type GM12878. **Supplementary figure 9**. The enrichment analysis on the allele-specific compartments in the cell line GM12878. (A) Example showing the consistence between the allele-biased pattern of compartment and allele-biased pattern of H3K4me1. In the chromatin region denoted by two black arrows, the maternal and paternal compartments are identified as B and A respectively, while the H3K4me1 signals are paternally biased in this region. (B) The statistical analyses on allele-specific compartments. The active epigenomic signals H3K4me1, H3K4me3 and H3K27ac were selected as representatives. In this calculation, the maternal and paternal compartment A are combined for the comparison to those of compartment B. **Supplementary figure 10**. The correlation analyses between allele-specific chromatin loops and allele-specific CTCF and Rad21 binding sites. (A) Examples showing the consistence between the allele-biased patterns of chromatin loops and allele-biased patterns of CTCF or Rad21 binding sites. In the left subfigure, the right anchor of chromatin loop shows higher Rad21 binding signals in paternal chromosome than that in maternal chromosome, consistent with the allelic direction of chromatin loop. In the right subfigure, the right anchors of the two allele-specific chromatin loops also show allelic biases in CTCF or Rad21 binding site to some extent. The solid circles denote the called chromatin loops and dashed circles denote the failure of loop calling. (B) Correlation analyses. The x-axis and y-axis denote the allelic ratios of chromatin loop and CTCF/Rad21 binding site respectively, by using the number of maternally mapped reads dividing the number of maternally plus paternally mapped reads.

## Data Availability

The Hi-C data sets of human cell line GM12878 and the mouse ICM were downloaded from NCBI with accession numbers GSE63525 [2] and GSE82185 [19] respectively. The Hi-C data set of mouse embryos at day 7.5 (E7.5) was downloaded from the Genome Sequence Archive in BIG Data Center in Chinese Academy of Sciences with the accession number PRJCA000241 [20]. The maternal and paternal genomes of cell line GM12878 were obtained from 1000 Genome Project [31]. The individual genomes of mouse strains DBA/2 and PWK/PhJ were obtained from mouse genome project (https://www.sanger.ac.uk/science/data/mouse-genomes-project) [32], and the maternal and paternal genomes of mouse cell types E7.5 and ICM were built by using corresponding SNPs. The ChIP-Seq data and corresponding peaks were downloaded from ENCODE portal (https://www.encodeproject.org/) [33] with the following identifiers: ENCSR000AKF, ENCSR000AKA, ENCSR000AKC, ENCSR000AKB, ENCSR000EAC.

## Abbreviations

3D: Three dimensional
ICM: Inner cell masses from blastocyst
E7.5: Mouse embryos at 7.5 day
SNP: Single nucleotide polymorphism
VC: Vanilla coverage
PCC: Pearson correlation coefficient
PC1: The first principal component
PC2: The second principal component
DI: Directionality index
