# Supplementary material for: HiCHap: a package to correct and analyze the diploid Hi-C data
Source: BMC Genomics. 2020 Oct 27;21:746. doi: 10.1186/s12864-020-07165-x (PMC7590616; doi:10.1186/s12864-020-07165-x)
Supplement: Supplementary file 1 — Additional file 1 : Supplementary figure 1. Two-end assigned contact matrices. (A) Examples showing the sparseness of two-end assigned contact matrices. The contact matrices in human cell line GM12878 are sparser than those in the E7.5 and ICM due to its lower SNP density. E7.5 and ICM denote the mouse embryos (day 7.5) crossed by C57BL/6 and DBA/2 and mouse inner cell masses from blastocyst crossed by C57BL/6 and PWK/PhJ respectively. (B) The intra-haplotype contacts dominate the two-end assigned contacts in all three data sets. Supplementary figure 2. Examples of different kinds of gaps diploid Hi-C contact maps defined in HiCHap. The heatmaps are from the cell line GM12878. (A) Gap bins defined in asymmetrical matrix. (B) Compartment gaps defined in compartment identification. PC1 denotes the first principle component. (C) Boundary gaps defined in boundary calling. (D) Loop gap defined in loop calling. The loop gap here is the zero-value contact around given circle. Supplementary figure 3. Evaluation on the parameter robustness in the SNP-bias correction. (A) Matrix similarities among different percentile values used in the definition of gap bin. (B) Matrix similarities among different threshold values used in the definition of gap bin. (C) Matrix similarities among different percentile values used in the definition of SNP-bias correction factor. For each parameter at given resolution, the diploid contact matrices were generated by using five different values, in which the default values for the other two parameters were used. The similarities among the five maternal contact matrices were calculated chromosome-by-chromosome by using HiCRep (see Materials and methods), and the X chromosomes were excluded from the calculations. The average values were shown in the subfigures. Supplementary figure 4. Replicate reproducibility for the selected eigenvectors in principal component analysis. Haploid reproducibility was calculated by using the first eigenvectors derived [file 12864_2020_7165_MOESM1_ESM.pdf]

# HiCHap: a package to correct and analyze the diploid Hi-C data

Han Luo[1,#], Xinxin Li[1,#], Haitao Fu[1], Cheng Peng[2,*]

[1]Hubei Key Laboratory of Agricultural Bioinformatics, College of Informatics, Huazhong Agricultural University, Wuhan 430070, China.

[2]Center for Life Sciences, College of Life Sciences, Yunnan University, Kunming 650500, China.

#Co-first authors. Han Luo and Xinxin Li contributed equally to this work.
*To whom correspondence should be addressed: chengpeng@ynu.edu.cn

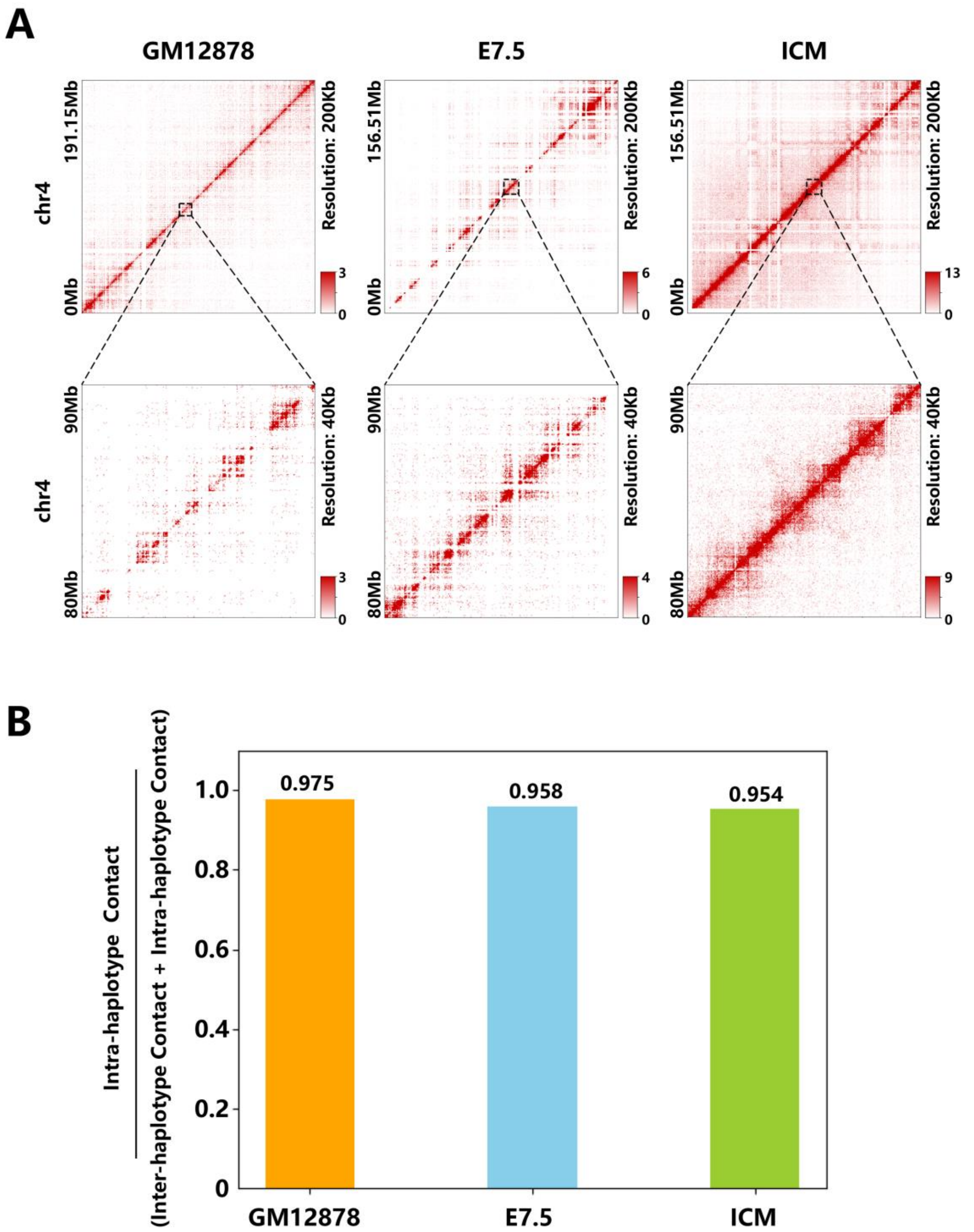


Supplementary figure 1. Two-end assigned contact matrices. (A) Examples showing the sparseness of two-end assigned contact matrices. The contact matrices in human cell line GM12878 are sparser than those in the E7.5 and ICM due to its lower SNP density. E7.5 and ICM denote the mouse embryos (day 7.5) crossed by C57BL/6 and DBA/2 and mouse inner cell masses from blastocyst crossed by C57BL/6 and PWK/PhJ respectively. (B) The intra-haplotype contacts dominate the two-end assigned contacts in all three data sets.

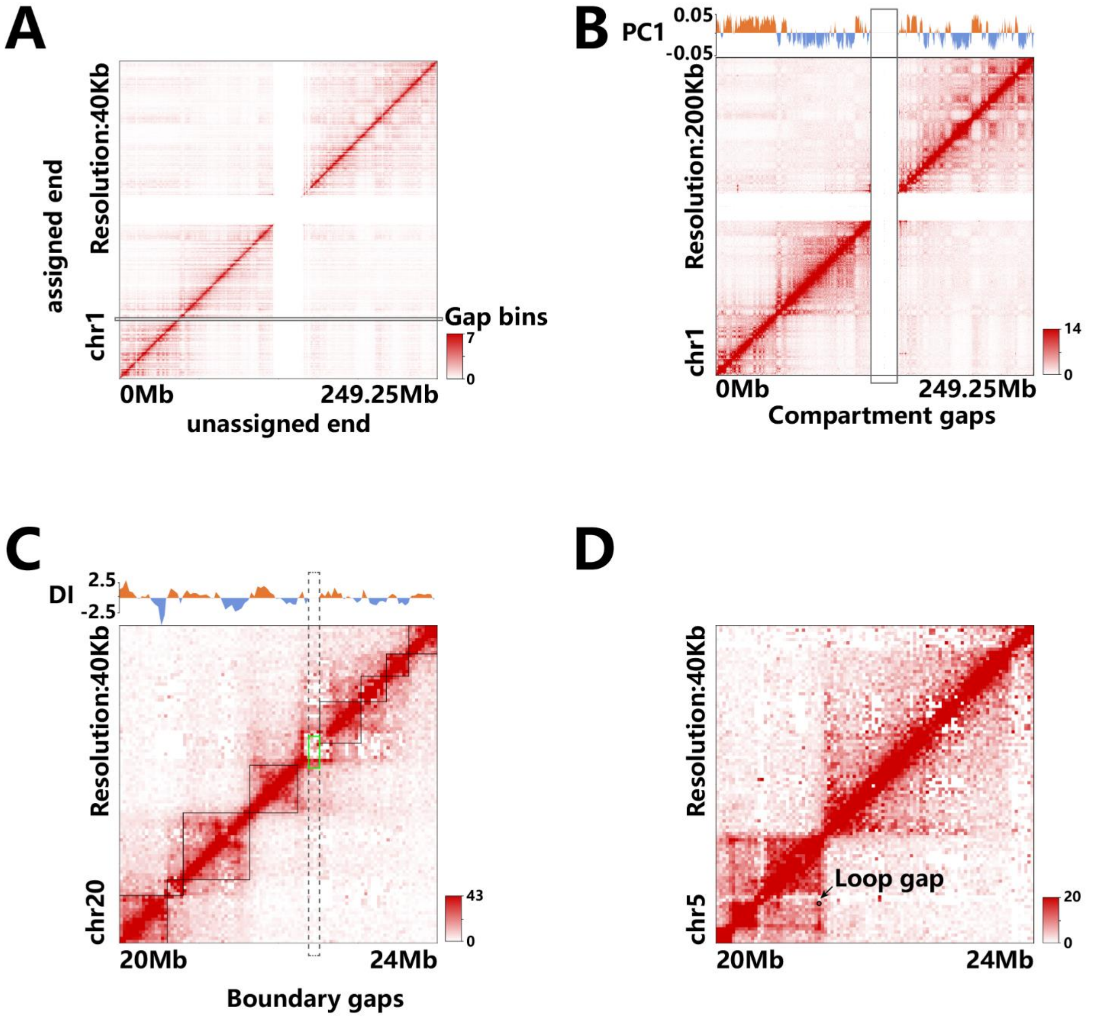


Supplementary figure 2. Examples of different kinds of gaps diploid Hi-C contact maps defined in HiCHap. The heatmaps are from the cell line GM12878. (A) Gap bins defined in asymmetrical matrix. (B) Compartment gaps defined in compartment identification. PC1 denotes the first principle component. (C) Boundary gaps defined in boundary calling. (D) Loop gap defined in loop calling. The loop gap here is the zero-value contact around given circle.

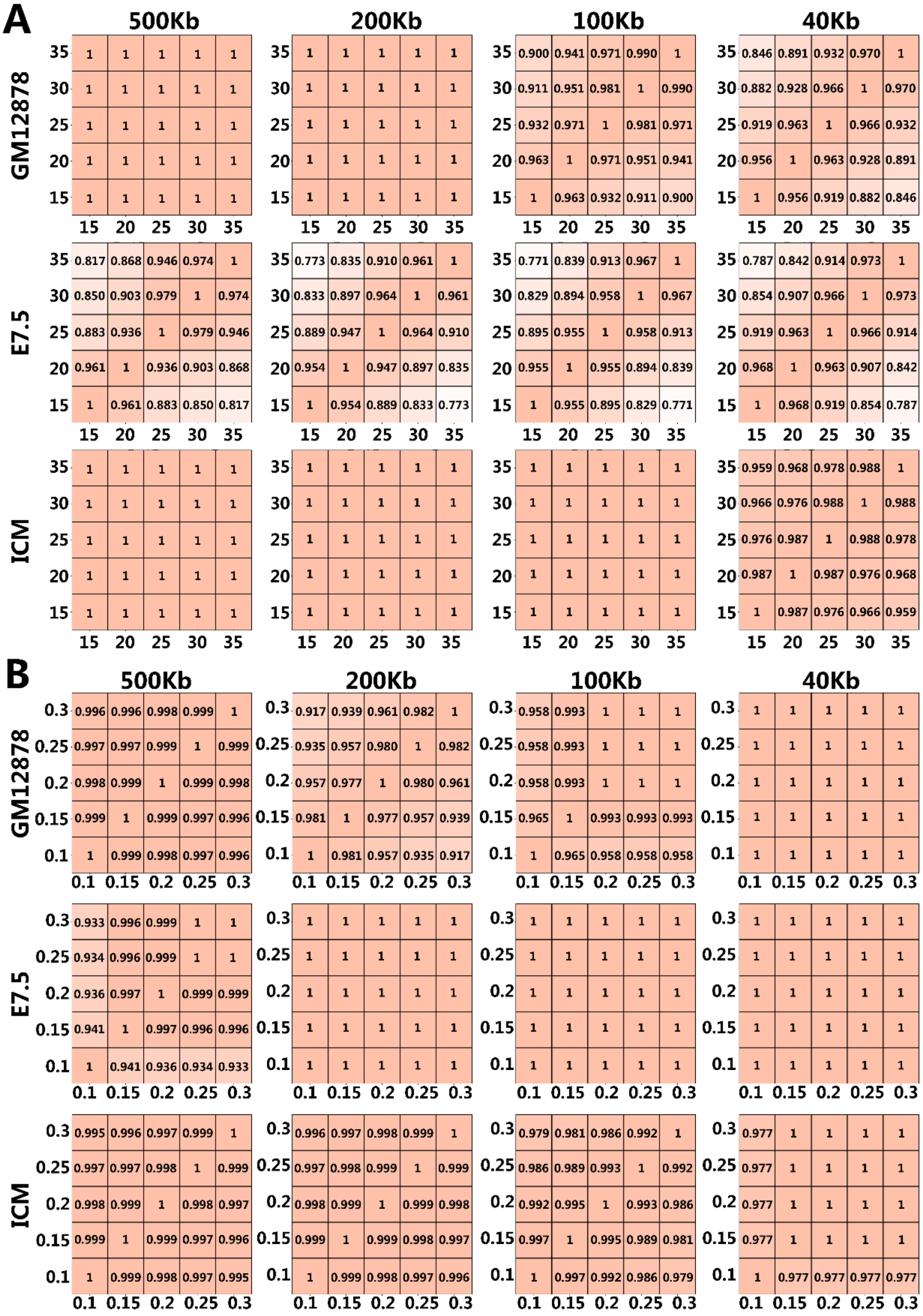
A
500Kb
200Kb
100Kb
40Kb
GM12878
E7.5
ICM
B
500Kb
200Kb
100Kb
40Kb
GM12878
E7.5
ICM

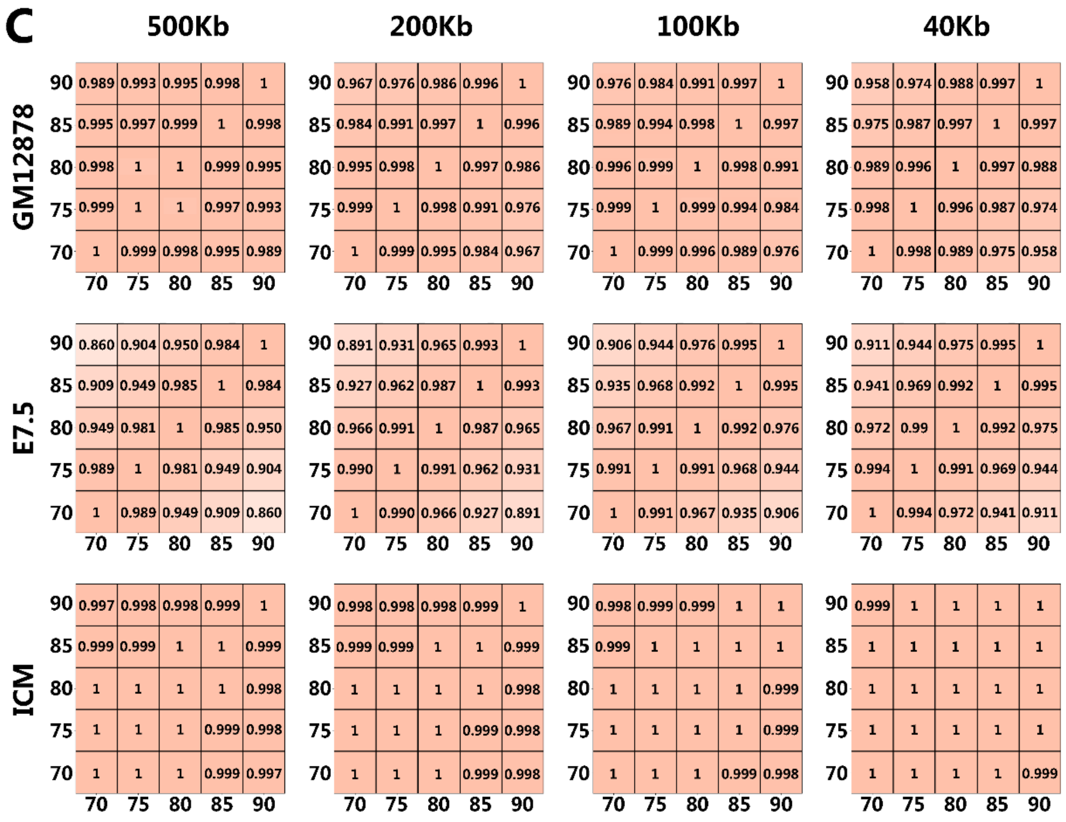


Supplementary figure 3. Evaluation on the parameter robustness in the SNP-bias correction. (A) Matrix similarities among different percentile values used in the definition of gap bin. (B) Matrix similarities among different threshold values used in the definition of gap bin. (C) Matrix similarities among different percentile values used in the definition of SNP-bias correction factor. For each parameter at given resolution, the diploid contact matrices were generated by using five different values, in which the default values for the other two parameters were used. The similarities among the five maternal contact matrices were calculated chromosome-by-chromosome by using HiCRep (see Materials and methods), and the X chromosomes were excluded from the calculations. The average values were shown in the subfigures.

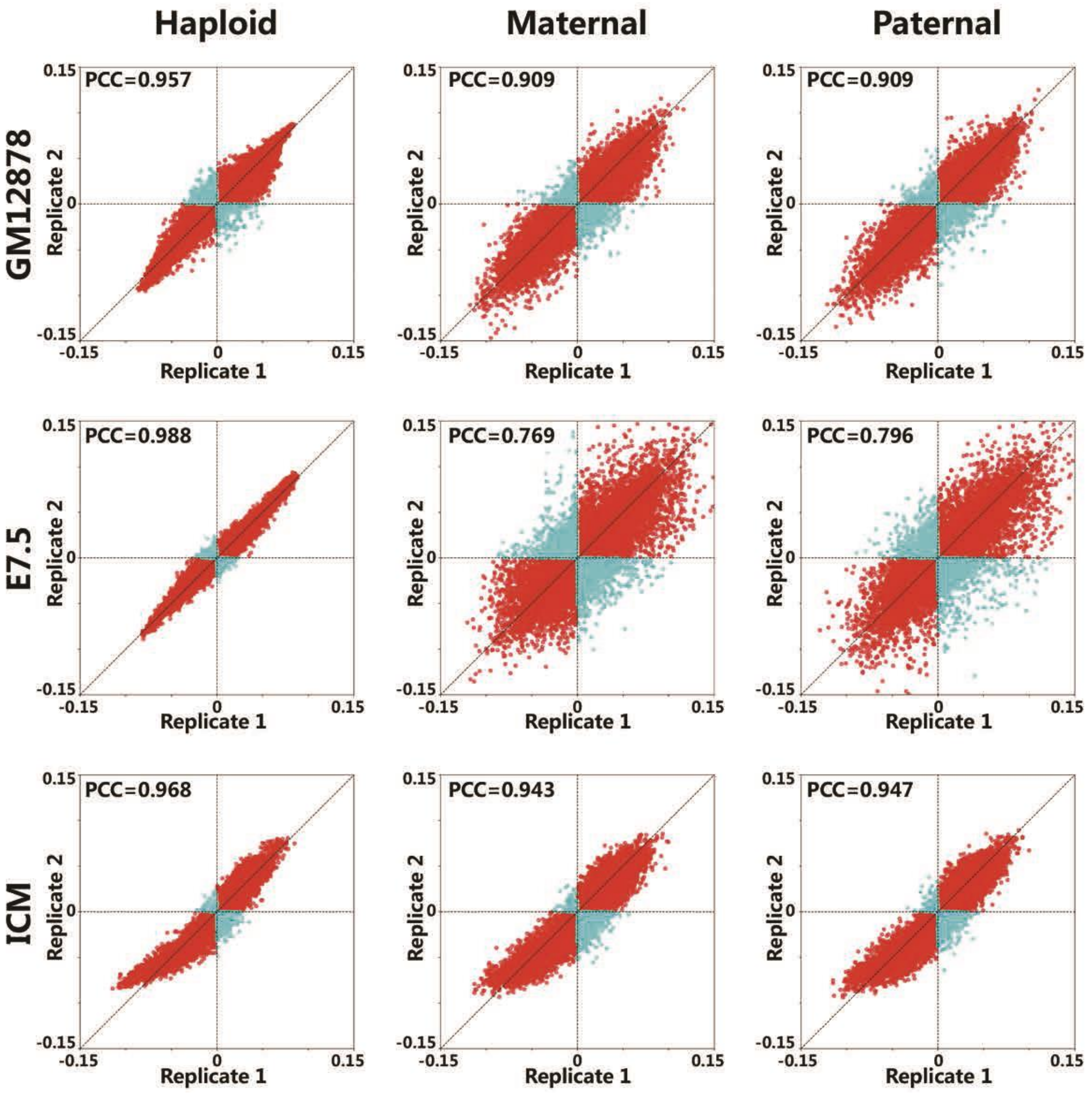


Supplementary figure 4. Replicate reproducibility for the selected eigenvectors in principal component analysis. Haploid reproducibility was calculated by using the first eigenvectors derived from two replicated haploid contact maps. The maternal and paternal reproducibility was calculated in the same way. In the cell line GM12878 and cell type ICM, the first eigenvectors were selected for all chromosomes. In cell type E7.5, the second eigenvectors were selected for five chromosomes and the first eigenvectors were selected for the rest ones. The light green points indicate that the two eigenvector entries of the same chromatin bin can exhibit the signed change between two biological replicates, suggesting the necessity to account for the intrinsic variations when calculating allele-specific compartments (or compartmental transitions). Actually, the variations are higher in maternal and paternal contact maps than those in haploid contact maps. PCC denotes Pearson correlation coefficient.

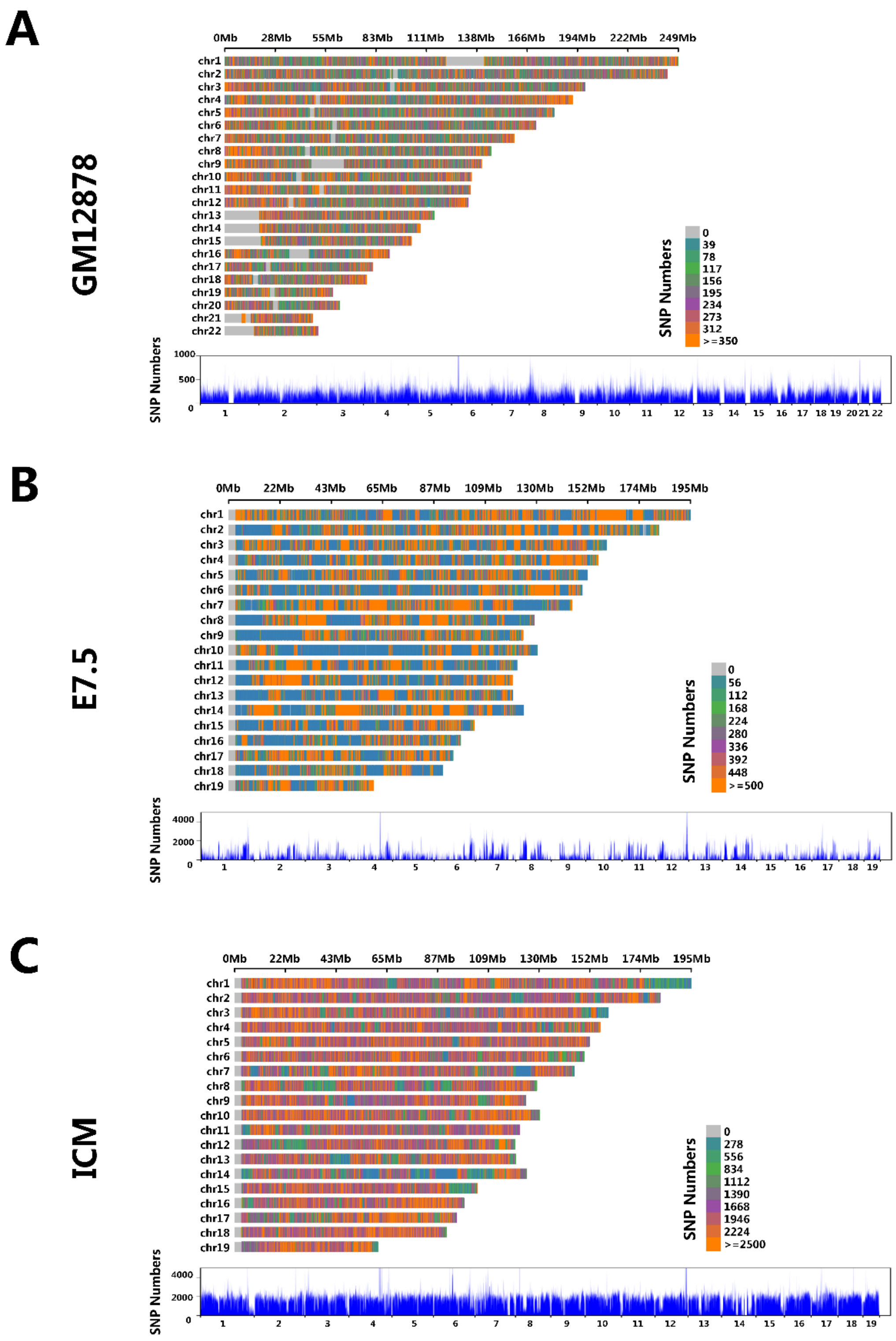


Supplementary figure 5. The SNP density distributions on chromosomes at the 200 kb resolution. (A) to (C) show the results in the cell line GM12878, cell type E7.5 and cell type ICM respectively. Compared to GM12878 and E7.5, the cell type ICM has the highest SNP density and relatively balanced SNP number in each chromosomal bin. By contrast, the cell type E7.5 exhibits the most variable SNP density in chromosomal bins.

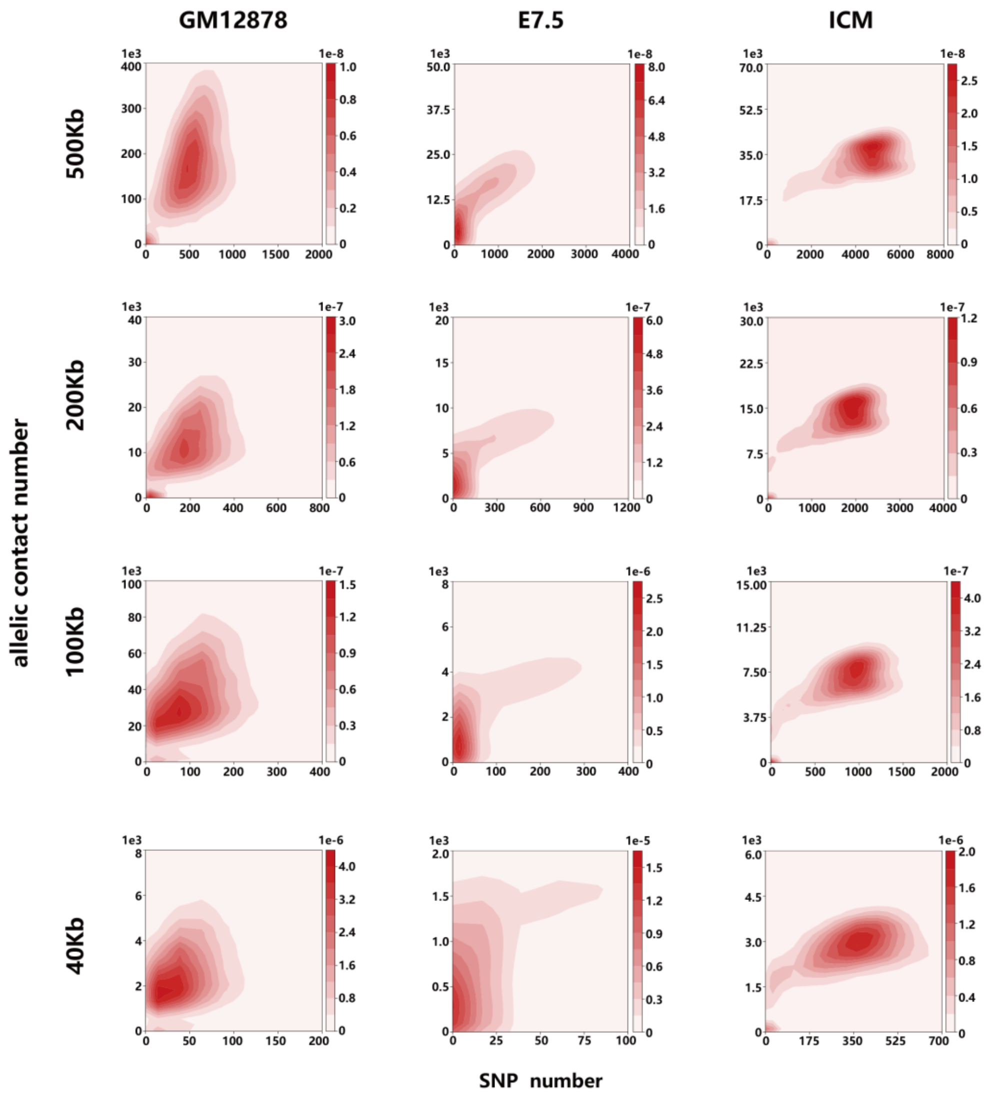


Supplementary figure 6. The relationship between allelic contact number and SNP number in cell line GM12878, cell type E7.5 and cell type ICM. The allelic contact denotes the contact which is assigned to be maternal or paternal one. In all subfigures, the x-axis and y-axis represent SNP number and allelic contact number respectively.

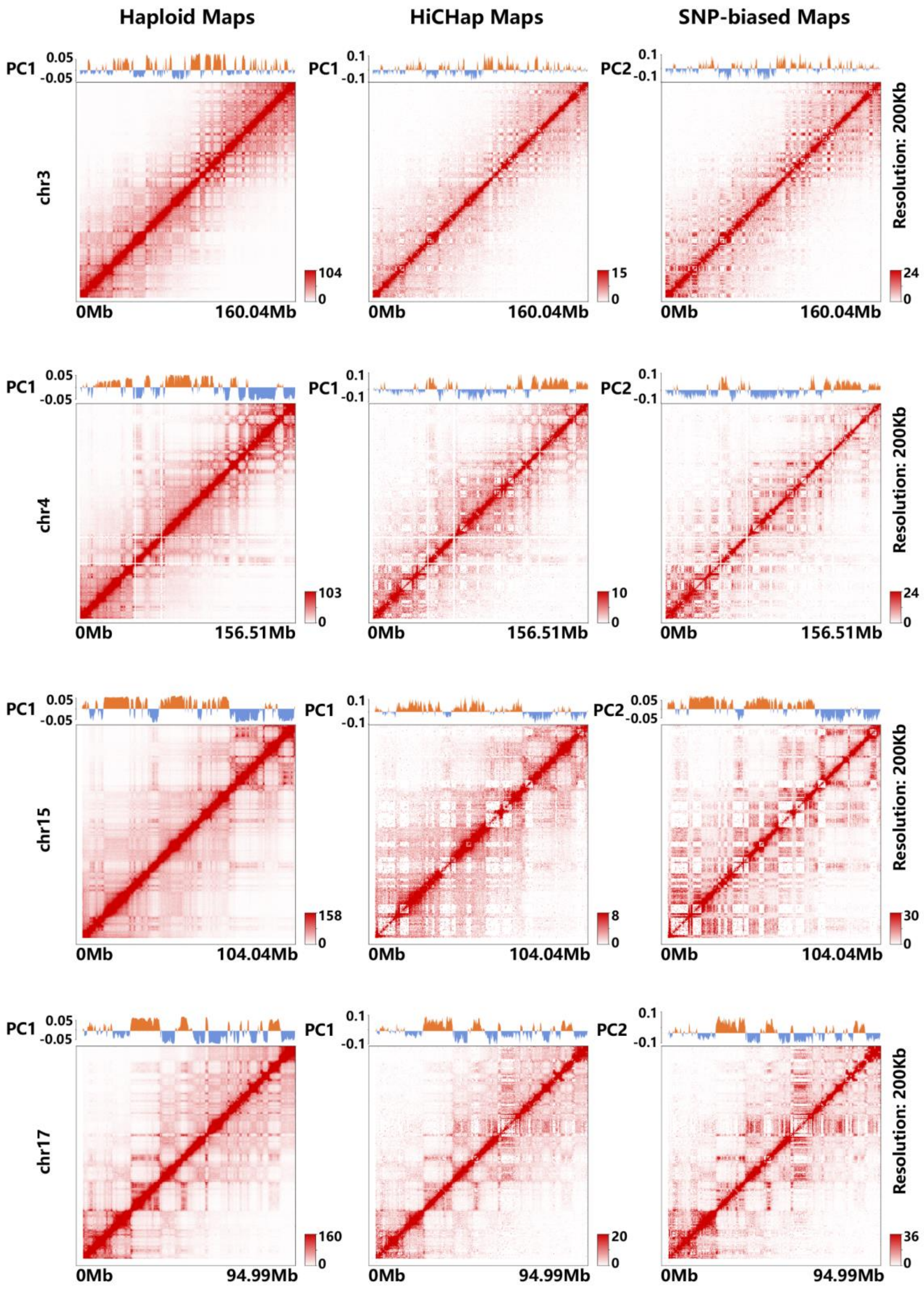


Supplementary figure 7. The examples showing the differences in the used principle component number between HiCHap contact maps and SNP-biased contact maps in the cell type E7.5 at 200 kb resolution. PC1 and PC2 denote the first and second principle component respectively.

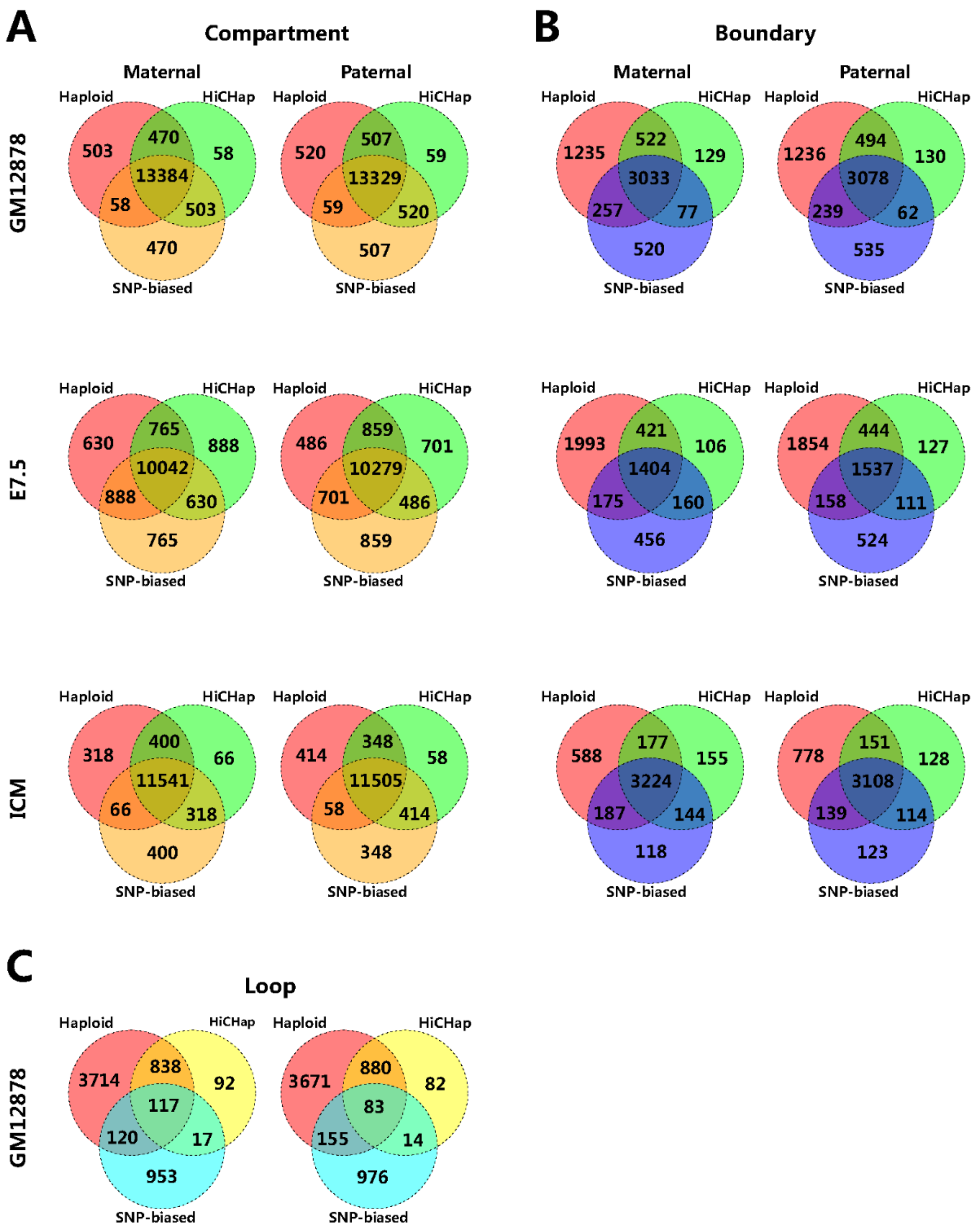


Supplementary figure 8. The consistence of compartments, topological boundaries and chromatin loops among haploid contact maps, HiCHap contact maps and SNP-biased contact maps. (A) Compartment. Compared to SNP-biased contact maps, the A/B compartments derived from HiCHap contact maps show much better consistence with those derived from haploid contact maps in cell types GM12878 and ICM. In cell type E7.5, the consistence level is comparable between HiCHap contact maps and SNP-biased contact maps. (B) Boundary. The boundaries derived from HiCHap cotant maps show much better consistence with those derived from haploid contact maps in cell types GM12878 and E7.5, and comparable consistence in cell type ICM. (C) Chromatin loop. The trend in chromatin loop is similar to those in compartment and boundary in cell type GM12878.

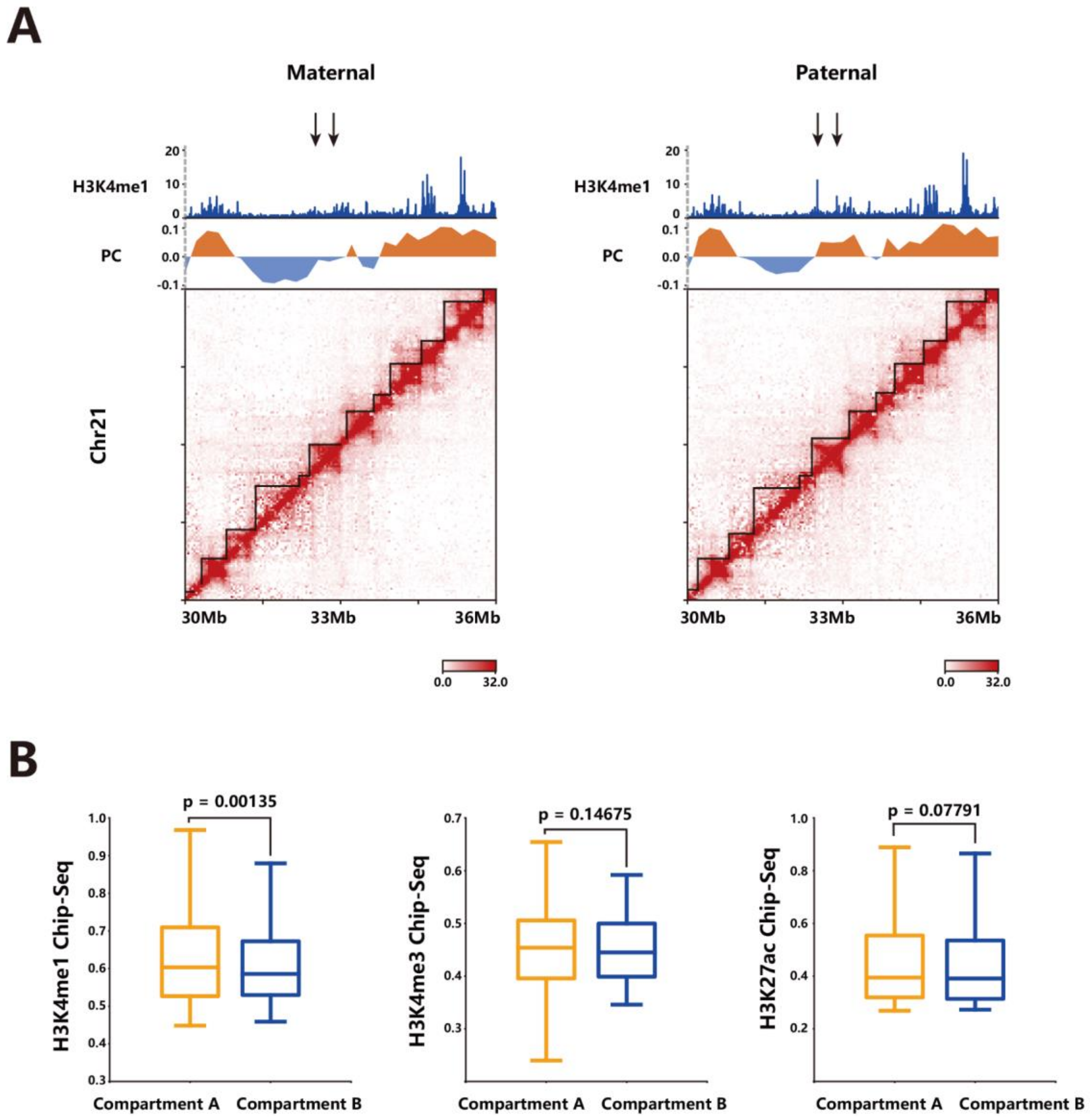


Supplementary figure 9. The enrichment analysis on the allele-specific compartments in the cell line GM12878. (A) Example showing the consistence between the allele-biased pattern of compartment and allele-biased pattern of H3K4me1. In the chromatin region denoted by two black arrows, the maternal and paternal compartments are identified as B and A respectively, while the H3K4me1 signals are paternally biased in this region. (B) The statistical analyses on allele-specific compartments. The active epigenomic signals H3K4me1, H3K4me3 and H3K27ac were selected as representatives. In this calculation, the maternal and paternal compartment A are combined for the comparison to those of compartment B.

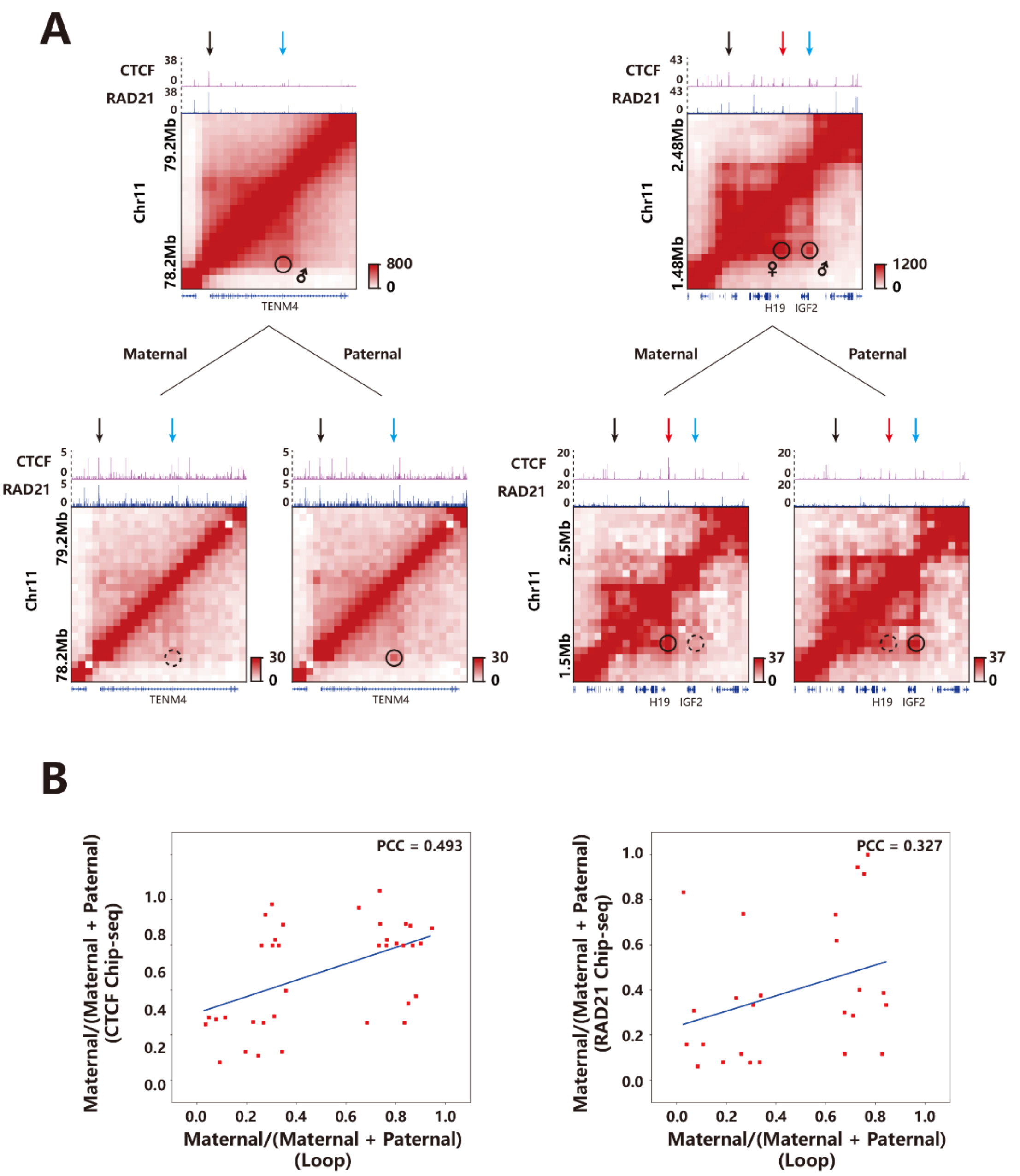


Supplementary figure 10. The correlation analyses between allele-specific chromatin loops and allele-specific CTCF and Rad21 binding sites. (A) Examples showing the consistence between the allele-biased patterns of chromatin loops and allele-biased patterns of CTCF or Rad21 binding sites. In the left subfigure, the right anchor of chromatin loop shows higher Rad21 binding signals in paternal chromosome than that in maternal chromosome, consistent with the allelic direction of chromatin loop. In the right subfigure, the right anchors of the two allele-specific chromatin loops also show allelic biases in CTCF or Rad21 binding site to some extent. The solid circles denote the called chromatin loops and dashed circles denote the failure of loop calling. (B) Correlation analyses. The x-axis and y-axis denote the allelic ratios of chromatin loop and CTCF/Rad21 binding site respectively, by using the number of maternally mapped reads dividing the number of maternally plus paternally mapped reads.
